# The impact of metabolic stressors on mitochondrial homeostasis in a renal epithelial cell model of methylmalonic aciduria

**DOI:** 10.1038/s41598-023-34373-8

**Published:** 2023-05-11

**Authors:** Anke Schumann, Marion Brutsche, Monique Havermans, Sarah C. Grünert, Stefan Kölker, Olaf Groß, Luciana Hannibal, Ute Spiekerkoetter

**Affiliations:** 1 Department of General Paediatrics, Adolescent Medicine and Neonatology, Medical Center-University of Freiburg, Faculty of Medicine, Mathildenstr. 1, 79106 Freiburg, Germany; 2 Institute of Neuropathology, Faculty of Medicine, Medical Center, University of Freiburg, Freiburg, Germany; 3 Division of Neuropediatrics and Pediatric Metabolic Medicine, Center for Child and Adolescent Medicine, University Hospital Heidelberg, Heidelberg, Germany; 4 Department of General Paediatrics, Adolescent Medicine and Neonatology, Laboratory of Clinical Biochemistry and Metabolism, Medical Center, University of Freiburg, Faculty of Medicine, Freiburg, Germany

**Keywords:** Diseases, Medical research, Molecular medicine

## Abstract

Methylmalonic aciduria (MMA-uria) is caused by deficiency of the mitochondrial enzyme methylmalonyl-CoA mutase (MUT). MUT deficiency hampers energy generation from specific amino acids, odd-chain fatty acids and cholesterol. Chronic kidney disease (CKD) is a well-known long-term complication. We exposed human renal epithelial cells from healthy controls and MMA-uria patients to different culture conditions (normal treatment (NT), high protein (HP) and isoleucine/valine (I/V)) to test the effect of metabolic stressors on renal mitochondrial energy metabolism. Creatinine levels were increased and antioxidant stress defense was severely comprised in MMA-uria cells. Alterations in mitochondrial homeostasis were observed. Changes in tricarboxylic acid cycle metabolites and impaired energy generation from fatty acid oxidation were detected. Methylcitrate as potentially toxic, disease-specific metabolite was increased by HP and I/V load. Mitophagy was disabled in MMA-uria cells, while autophagy was highly active particularly under HP and I/V conditions. Mitochondrial dynamics were shifted towards fission. Sirtuin1, a stress-resistance protein, was down-regulated by HP and I/V exposure in MMA-uria cells. Taken together, both interventions aggravated metabolic fingerprints observed in MMA-uria cells at baseline. The results point to protein toxicity in MMA-uria and lead to a better understanding, how the accumulating, potentially toxic organic acids might trigger CKD.

## Introduction

Methylmalonic aciduria (MMA-uria, #OMIM 251000) is caused by mutations in the mitochondrial enzyme methylmalonyl-CoA mutase (MUT)^1,2^. MUT catalyzes the final step in the anaplerotic pathway that forms succinyl-CoA from the breakdown of isoleucine, methionine, threonine and valine (IMTV), odd-chain fatty acids, and cholesterol. Biochemically, MMA-uria is characterized by elevation of propionyl-CoA, propionylcarnitine (C3), 3-OH-propionate, methylcitrate (MC), and methylmalonic acid (MMA). MMA-uria patients face a life-long risk of life-threatening metabolic crisis in times of catabolism or excessive protein intake^1^. Multi-organ dysfunction, particularly neurological impairment and chronic kidney disease (CKD), are known long-term complications^1,3^. Residual MUT activity inversely correlates with biochemical and clinical severity of MMA-uria^3,4^. Metabolic therapy consists of avoidance of catabolism, IMTV-reduced diet, and carnitine supplementation to enhance the formation of non-toxic C3^1^.

Multi-organ damage is thought to be caused by mitochondrial dysfunction and oxidative stress (ROS) due to toxic metabolite-induced synergistic inhibition of key enzymes of mitochondrial energy metabolism^5^. The toxicity of propionate metabolism intermediates has been linked to neurological impairment^1,2^ and fibrotic reorganization in liver tissue^6^. The kidney is not only rich in mitochondria but due to transport processes also highly exposed to these toxic metabolites whose increasing abundance in metabolic instability might promote renal fibrosis and CKD. This notion is further supported by megamitochondria, decreased respiratory chain activity and glutathione concentrations in human and murine liver and kidney samples for MMA-uria^7,8^. In Mut-deficient mice, antioxidant treatment reduced ROS levels and renal epithelial damage^9^. We recently identified a link between renal epithelial damage, accumulation of ROS species and defective mitochondrial priming for mitophagy in MMA-uria. Reintroduction of MUT reversed the phenotype, indicating that intracellular signaling mechanism interfering with mitochondrial quality control are affected in MMA-uria^10^. Besides ATP production, mitochondria also orchestrate multiple signaling pathways such as redox homeostasis, inflammation, and apoptosis^10^, which are crucial to maintain kidney function. Given the complexity of potential interacting factors, the identification of the mechanisms altering mitochondrial networks and intracellular signaling in MMA-uria need further investigation^11^. Due to its unique function and early involvement, the understanding of renal pathophysiology could be key to unravel pathophysiological mechanisms in organic acidurias.

This study evaluates if and how protein load or precursor amino acids add additional stress to mitochondrial homeostasis in renal epithelial cells in MMA-uria and compares these results with a previous study in propionic aciduria (PA-uria)^12^. Here we provide evidence that metabolic challenge in these cells induces major alterations in mitochondrial metabolism and homeostasis, highlighting the importance of organ-specific subcellular studies to identify potentially targetable pathways amenable to pharmacological interventions.

## Results

### Protein load impacts on renal tubular function in MMA-uria

**Figure 1:**
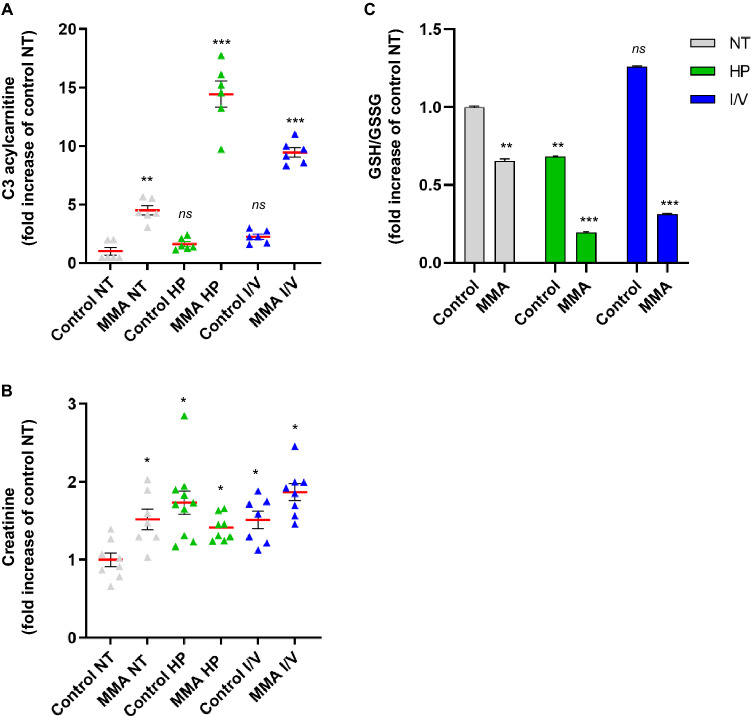
Protein load impacts on renal tubular function in MMA-uria. (**A**) Propionylcarnitine (C3) concentration in different treatment conditions: n = 9 per group. *ns* not significant. (**B**) LC–MS/MS quantification of creatinine concentrations n = 9 per group. (**C**) Quantitative LC–MS/MS analysis ratio of oxidized (GSSG) to reduced levels of glutathione (GSH). n = 9 per group. Different exposures are indicated as: *NT* normal treatment, *HP* high-protein treatment, *I/V* isoleucine/valine treatment. Control (control), MMA-uria patient cells (MMA). * as compared to control NT.

We exposed human renal tubular cells of healthy controls and MMA-uria patients with loss of MUT activity (*MUT*^0^, Table 1) to either high glucose (NT), low glucose and high protein (HP), or low glucose and high isovaline/valine (I/V) treatment to investigate the impact of these metabolic challenges on renal mitochondrial energy metabolism. We used (LC-)MS/MS to quantify the effects of these exposures on disease-specific metabolic markers (C3), renal function, and anti-oxidant defense. MMA-uria cells showed elevated levels of C3 under NT conditions (4.3-fold), which were potentiated especially by HP (14.5-fold) but also by I/V exposure (9.7-fold; Fig. 1A, Suppl. Table 1). Creatinine concentrations were in tendency higher in MMA-uria cells compared to control NT (Fig. 1B); however, protein load also increased creatinine concentrations in control cells. The glutathione system is one of most potent buffers against ROS. The ratio of reduced (GSH) to oxidized (GSSG) glutathione was reduced in MMA-uria cells under NT (0.65-fold; Fig. 1C) highlighting a compromised antioxidant system. HP (0.2-fold) and I/V (0.3-fold) exposure worsened the GSH/GSSG ratio and hence had a harmful impact on MMA-uria cells (Fig. 1C). Control cells showed a reduction for HP exposure, while the ratio was increased in I/V conditions.

**Table 1:** Enzymatic activity expressed in pmol × min^−1^ × mg^−1^ of methylmalonyl-CoA-mutase activity. Data expressed as mean ± SEM (n = 3). Table modified from^10^.

Cell line	Class	Change		Enzymatic activity
		Nucleotide	Amino acid	
Control_1	–	–	–	3403 ± 136
Control_2	–	–	–	7406 ± 336
Control_3	–	–	–	3738 ± 218
MMA_1	*MUT*^*0*^	c.607C>A; c.1105C>T	p.S288P;p.H386R	66 ± 18
MMA_2	*MUT*^*0*^	c.862T>C; c.862T>C	p.S288P;p.S288P	17 ± 10
MMA_3	*MUT*^*0*^	c.982C>T; c.982C>T	p.L328F;p.L328F	38 ± 25

### Impact of protein load on cellular viability and apoptosis

Next, we investigated the impact of various metabolic challenges on cellular viability and cell survival using FACS analysis. Cells were co-stained with Annexin V and 7AAD to distinguish between apoptotic and lytic cells. Double negative cells were considered viable.

**Figure 2:**
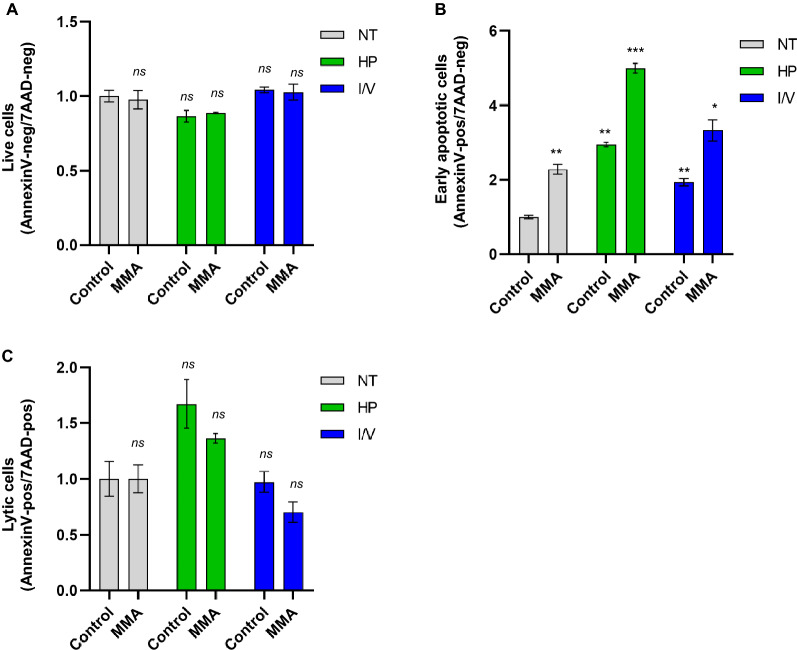
Apoptosis is activated in MMA-uria. (**A**) FACS analysis for Annexin V negative (Annexin V neg) and 7-amino-actinomycin D negative (7AAD neg) cells as read-out for cellular viability. (**B**) FACS analysis for Annexin V positive (Annexin V pos) and 7-amino-actinomycin D (7AAD)-negative cells as read-out for early apoptotic cells. (**C**) FACS analysis for Annexin V positive (Annexin V pos) and 7-amino-actinomycin D (7AAD)-positive cells as read-out for lytic cells. Different exposures are indicated as: *NT* normal treatment, *HP* high-protein treatment, *I/V* isoleucine/valine treatment. Control (control), MMA-uria patient cells (MMA). Data are expressed relative to control NT; n = 6 per group. * as compared to control NT.

Cellular viability was similar in all cell lines and treatment conditions as compared to control NT (Fig. 2A). Apoptosis was more pronounced in MMA-uria cells using NT (2.3-fold) and potentiated by HP (5-fold) and I/V (3.3-fold) exposure (Fig. 2B). However, HP and I/V treatment also affected apoptotic rates in control cells, but to a lesser extent. Cell lysis remained unchanged in control and MMA-uria cells (Fig. 2C), indicating that MMA-uria cells undergo programmed cell death.

### Mitochondrial stress response to protein load

We evaluated potential mitochondrial adaptation mechanisms to a compromised antioxidant system. We performed immunoblot analysis for the mitochondrial marker protein voltage dependent anion channel (VDAC).

**Figure 3:**
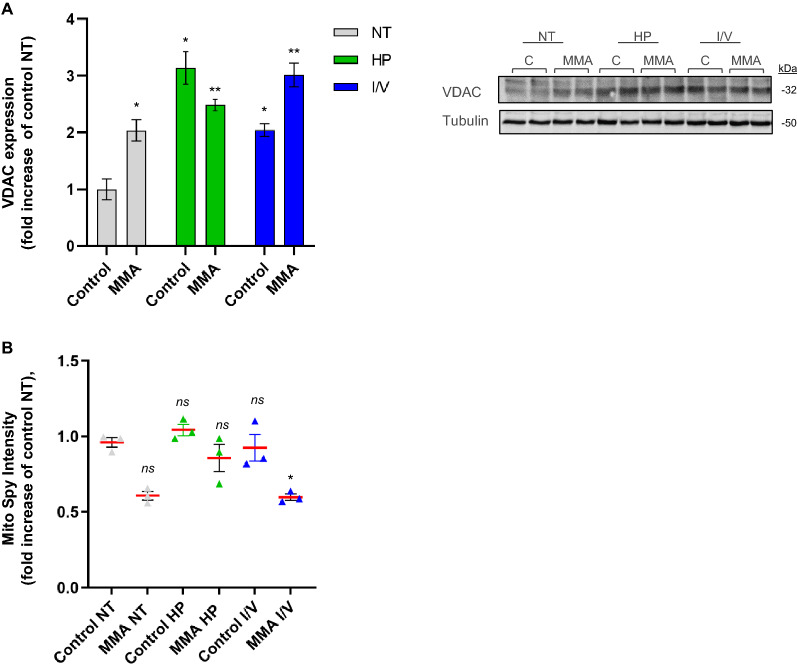
Mitochondrial stress response to protein load. (**A**) Immuno-blot analysis and quantification for voltage-dependent anion channel (VDAC) Tubulin was used as loading control. Different exposures are indicated as: *NT* normal treatment, *HP* high-protein treatment, *I/V* isoleucine/valine treatment. Control (control), MMA-uria patient cells (MMA-uria). (**B**) FACS analysis for MitoSpy intensity. Different exposures are indicated as: *NT* normal treatment, *HP* high-protein treatment, *I/V* isoleucine/valine treatment. Control (control), MMA-uria patient cells (MMA). Data are expressed relative to control NT; n = 50,000 cells per cell line. * as compared to control NT.

VDAC was up-regulated (2-fold) under NT conditions. Both HP (2.5-fold) and I/V exposure (3-fold) further pronounced VDAC expression compared to control NT pointing towards a need for mitochondrial compensation (Fig. 3A). While mitochondrial protein expression was up-regulated by metabolic stress, FACS analysis for MitoSpy, a mitochondrial dye independent of mitochondrial membrane potential, demonstrated signs of lowered mitochondrial numbers under NT conditions (0.7-fold), which were potentiated by I/V (0.5-fold) exposure (Fig. 3B).

### Mitochondrial energy metabolism is altered by protein load

**Figure 4:**
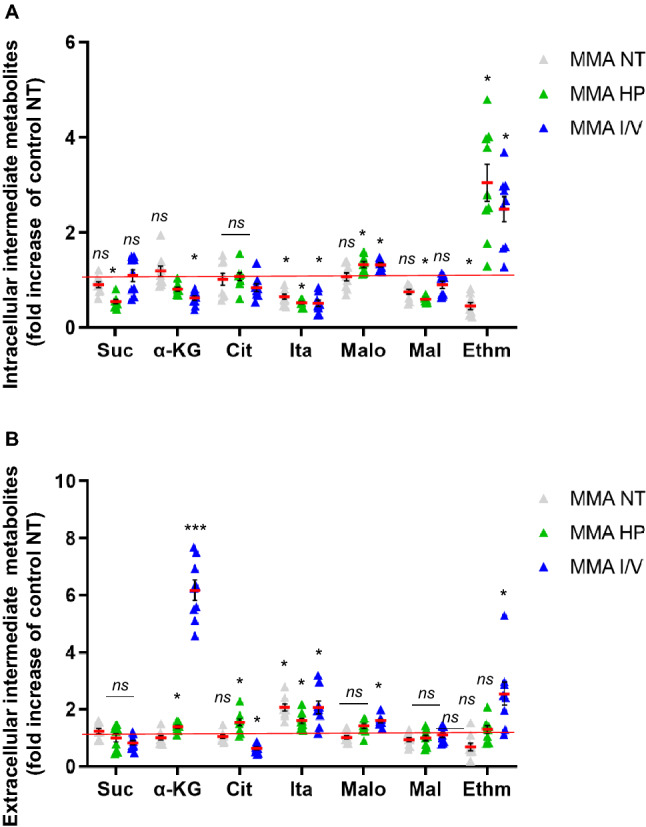
Mitochondrial energy metabolism is altered by protein load. Quantitative LC–MS/MS analysis of intermediate metabolites in the intracellular (**A**) and extracellular (**B**) compartment. Abbreviation of metabolites: *Suc* succinate, *α-KG* alpha-ketoglutarate, *Cit* citrate, *Ita* itaconate, *Malo* malonate, *Mal* malate, *Ethm* ethylmalonate. Different exposures are indicated as: *NT* normal treatment, *HP* high-protein treatment, *I/V* isoleucine/valine treatment. Control (control), MMA-uria patient cells (MMA). Data are expressed relative to control NT (red line); n = 9 per group. * as compared to control NT.

We investigated how the different exposures impact on TCA metabolites in MMA-uria. LC–MS/MS investigation revealed comparable intracellular succinate levels for NT and I/V exposure, while HP incubation led to succinate depletion by a factor of two (Fig. 4A, Suppl. Table 2A). Intracellular alpha-ketoglutarate concentration remained unchanged for NT conditions and was lowered by I/V exposure (0.6-fold; Fig. 4A, Suppl. Table 2A). Itaconate concentrations were markedly reduced in MMA-uria cells regardless of the medium conditions (0.6- and 0.5-fold; Fig. 4A, Suppl. Table 2A). Malonate, a known inhibitor of succinate dehydrogenase, was elevated using HP (1.3-fold) and I/V (1.3-fold) while malate levels were lowered (Fig. 4A, Suppl. Table 2A). Extracellular levels of alpha-ketoglutarate were elevated under HP (1.4-fold) and I/V (6.3-fold) conditions (Fig. 4B, Suppl. Table 2B). Itaconate and malonate export were increased in MMA cells in all experimental settings (Fig. 4B, Suppl. Table 2B).

**Figure 5:**
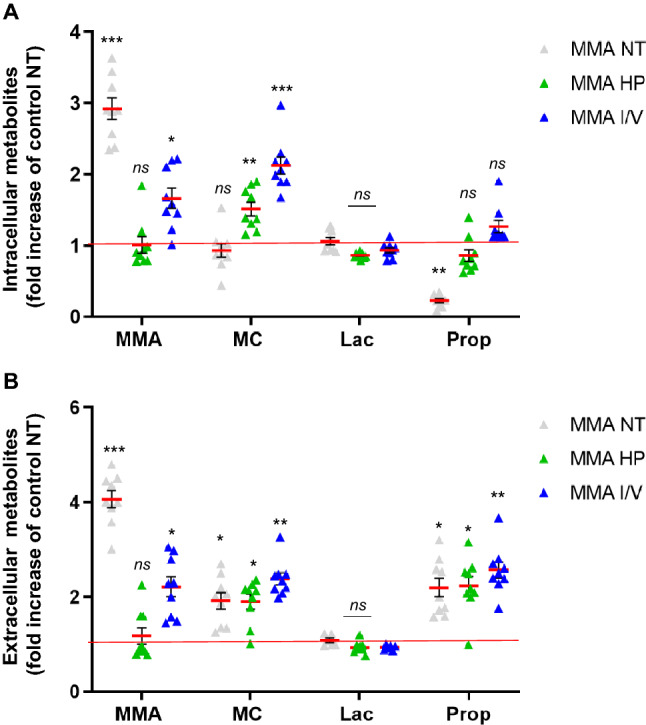
Modification of MMA-uria associated metabolites. Quantitative LC–MS/MS analysis of MMA-uria associated metabolites in the intracellular (**A**) and extracellular (**B**) compartment. Abbreviation of metabolites: *MMA* methylmalonic acid, *MC* methylcitrate, *Lac* lactate, *Prop* propionate. Different exposures are indicated as: *NT* normal treatment, *HP* high-protein treatment, *I/V* isoleucine/valine treatment. Control (control), MMA patient cells (MMA). Data are expressed relative to control NT (red line); n = 9 per group. * as compared to control NT.

We next investigated the impact of dietary modifications toxic metabolites. In the intracellular compartment, LC–MS/MS studies revealed threefold elevated levels of MMA under NT conditions (Fig. 5A, Suppl. Table 3A). MMA-uria cells exposed to HP showed MMA levels comparable to control cells under NT, while I/V exposure led to a 1.6-fold increase (Fig. 5A, Suppl. Table 3A). Both HP (1.5-fold) and I/V (2.1-fold) exposure led to an increase in MC levels (Fig. 5A, Suppl. Table 3A). Lactate concentrations were similar to control NT conditions. Propionate levels were not elevated, but in tendency higher under HP or I/V exposure than under NT (Fig. 5A, Suppl. Table 3A). Investigation of extracellular metabolites revealed that MMA is secreted by renal epithelial cells, when intracellular concentrations are high. This can especially be seen under NT conditions. HP and I/V exposure did not exacerbate MMA secretion (Fig. 5B, Suppl. Table 3B). MC was secreted in sufficient amounts to keep intracellular levels normal under NT. However, HP and I/V exposure led to an increase in MC export (1.9 and 2.5-fold) which did not seem to be sufficient to keep intracellular concentrations low (Fig. 5B, Suppl. Table 3B). Extracellular lactic acid levels remained comparable to control cells NT in all conditions. Propionic acid (PA) was exported in comparable amounts (Fig. 5B, Suppl. Table 3B).

### Metabolic profiling under different treatment conditions

**Figure 6:**
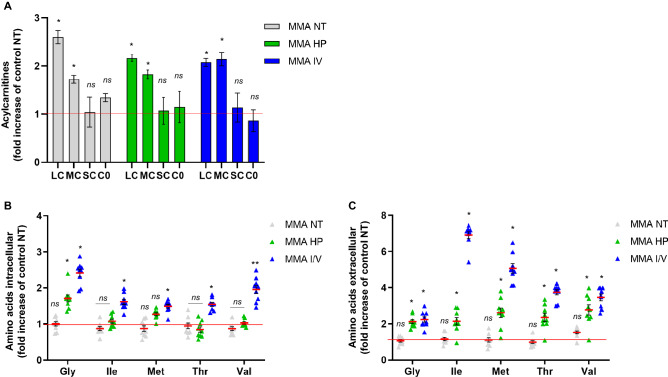
Metabolic profiling in different treatment conditions. (**A**) Acylcarnitine profile of cell lysates derived from control and MMA-uria patient cells (MMA-uria). *NT* normal treatment, *HP* high-protein treatment, *I/V* isoleucine/valine treatment. Data are expressed relative to control (red line). The acylcarnitines were summed up by the respective acyl-group chain-length: *LC* long-chain acylcarnitine (C12–C18); *MC* medium-chain acylcarnitine (C6–C10); *SC* short-chain acylcarnitine (C2–C5); *C0* free carnitine. n = 12 per group. (**B**) Amino acid profile [µmol/L] in cell lysates of control and MMA-uria patient cells. (**C**) Amino acid profile [µmol/L] in cell culture medium of control and MMA-uria patient cells. Different exposures are indicated as: *NT* normal treatment, *HP* high-protein treatment, *I/V* isoleucine/valine treatment. Control (control), MMA-uria patient cells (MMA). Data are expressed relative to control NT (red line); n = 9 per group. * as compared to control NT.

Next, we studied the impact of metabolic stressors on mitochondrial energy metabolism. We chose acylcarnitine profiling as read-out for the beta-oxidation pathway. Especially long- (LC) and medium-chain (MC) acylcarnitines accumulated in MMA-uria patient cells (Fig. 6A, Suppl. Table 1). LC-acylcarnitines were highest under NT conditions (2.5-fold elevated) and similarly high using HP (2.1-fold) and I/V (2.1-fold). I/V exposure increased MC-acylcarnitine concentrations (2.1-fold) and concomitantly lowered free carnitine (0.7-fold, Fig. 6A, Suppl. Table 1). In a next step, we analyzed the amino acid profile. As a proof of concept, isoleucine and valine concentrations were highest in the I/V treated cells (Fig. 6B,C, Suppl. Table 4A). Glycine levels were elevated under HP exposure (1.6-fold as compared to control NT conditions) and even higher under I/V exposure (2.3-fold). Methionine concentrations were also elevated under HP (1.2-fold) and I/V (1.6-fold) conditions, while threonine was only increased under I/V conditions (Fig. 6B, Suppl. Table 4A). Indigestible amino acids and glycine were exported under HP and I/V conditions (Fig. 6C).

### Mitochondrial quality control is affected by protein load

Having identified potentially toxic effects of both exposures and based on our previous results^10^, we studied whether different metabolic stressors further interfere with mitochondrial quality control systems, namely mitophagy and autophagy. We chose PTEN induced kinase 1 (PINK1), the initiator of mitophagy as marker protein for organelle-specific degradation and SQSTM1, as read-out for autophagy.

**Figure 7:**
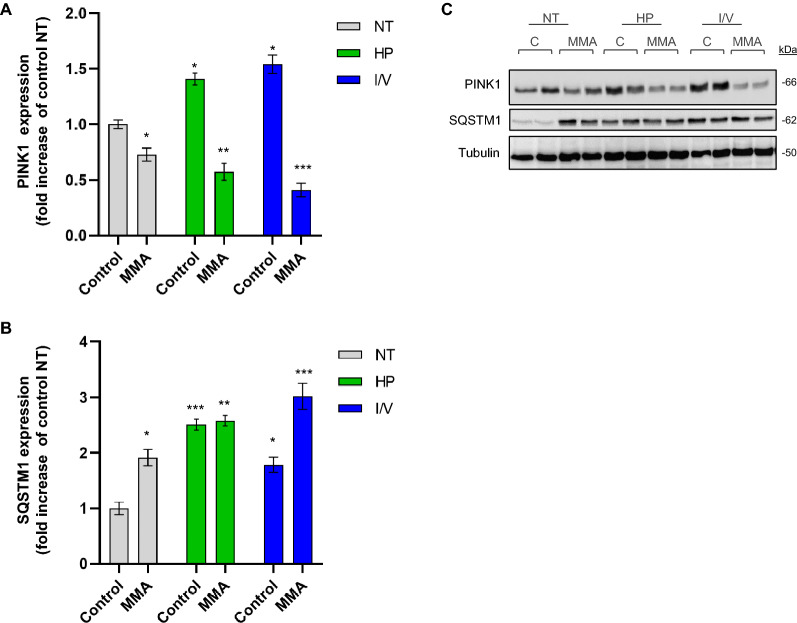
Mitochondrial quality control is affected by protein load. (**A**) Quantification of immuno-blot analysis for PINK1. (**B**) Quantification of immuno-blot analysis for SQSTM1. (**C**) Immuno-blot analysis for PINK1 and SQSTM1. Tubulin was used as loading control. Different exposures are indicated as: *NT* normal treatment, *HP* high-protein treatment, *I/V* isoleucine/valine treatment. Control (control), MMA-uria patient cells (MMA). Data are expressed relative to control NT; n = 9 per group. * as compared to control NT.

Confirming previous results, we found mitophagy to be downregulated in MMA-uria cells under NT conditions (0.7-fold; Fig. 7A,C)^10^. HP (0.6-fold) and particularly I/V treatment (0.4-fold; Fig. 7A,C) further lowered PINK1 expression (Fig. 7A,C). Both exposures led to a significant increase of PINK1 in control cells, suggesting a substantial metabolic challenge of these exposures. SQSTM1 was up-regulated in MMA-uria cells under NT conditions. HP treatment led to a 2.6-fold increase while I/V treatment led to a further rise (3-fold) as compared to control cells NT (Fig. 7B,C).

### Mitochondrial fission and fusion are altered in MMA-uria

In times of metabolic and environmental stress, mitochondrial fission and fusion are crucial to maintain a functional mitochondrial network. The processes are tightly connected with mitochondrial quality control. Fission, promoted by GTPase dynamin-related protein 1 (Drp1), facilitates separation of dysfunctional mitochondria and assists mitophagy. Fusion, regulated by OPA1 expression, results in the formation of interconnected mitochondrial networks and is furthermore associated with apoptosis.

**Figure 8:**
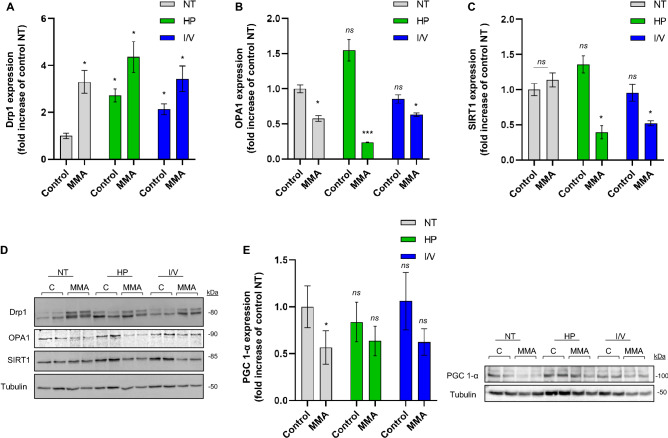
Mitochondrial fission and fusion is altered in MMA-uria. (**A**) Quantification of immuno-blot analysis for Drp1. (**B**) Quantification of immuno-blot analysis for OPA1. (**C**) Quantification of immuno-blot analysis for SIRT1. (**D**) Immuno-blot analysis for Drp1, OPA1 and SIRT1. Tubulin was used as loading control. (**E**) Immuno-blot analysis for peroxisome proliferator-activated receptor *γ* coactivator 1 *α* (PGC-1*α*). Tubulin was used as loading control. Different exposures are indicated as: *NT* normal treatment, *HP* high-protein treatment, *I/V* isoleucine/valine treatment. Control (control), MMA-uria patient cells (MMA). Data are expressed relative to control NT; n = 9 per group. * as compared to control NT.

MMA-uria cells showed a strong tendency towards mitochondrial fission in NT conditions as evidenced by elevated Drp1 levels (3.3-fold elevation). This effect was even more pronounced in HP (4.3-fold increase) and I/V (3.5-fold) conditions (Fig. 8A,D). OPA1 expression was comparably reduced in MMA-uria cells in NT (0.6-fold) and I/V conditions (Fig. 8B,D) and even more pronounced in HP (0.2-fold) exposure (Fig. 8B,D).

We next addressed the activation of renal protection mechanisms. We investigated SIRT1 expression, which has been related to cytoprotection by modulating inflammation, apoptosis and fibrotic remodeling^13^. SIRT1 expression was comparably high in control and MMA-uria cells in NT conditions. While HP and I/V exposure leads to an increase in SIRT1 levels in control cells, MMA cells show markedly reduced SIRT1 expression, pointing to insufficient activation of self-protection (Fig. 8C,D). Immuno-blotting for peroxisome proliferator-activated receptor *γ* coactivator 1*α* (PGC-1*α*) revealed reduced expression levels of this important regulator of mitochondrial biogenesis in MMA-uria cell lines (Fig. 8E).

## Discussion

The kidney is one of the most energy-consuming organs of the human body. Renal tubular cells are equipped with a large number of mitochondria to perform energy-dependent transport processes^14^. Mitochondrial impairment leads to decreased ATP production, organ damage and has been associated with onset and progression of CKD^14,15^. Disturbed mitochondrial function and defective mitochondrial priming for degradation has been linked renal epithelial damage in MMA-uria by our group^10^.

We investigated the effect of exposing renal epithelial MMA-uria cells to high protein and disease-associated amino acids to study the effect on mitochondrial energy metabolism and homeostasis. The aim was to elucidate pathomechanisms underlying CKD and to identify novel therapeutic targets. We furthermore compared mechanistic differences and similarities between MMA-uria and PA-uria, two organic acidurias caused by deficiency of mitochondrial enzymes catalyzing the final steps of the anaplerotic propionate pathway. In contrast to MMA-uria, CKD only occurs as late complication in PA-uria^15,16^.

**Figure 9:**
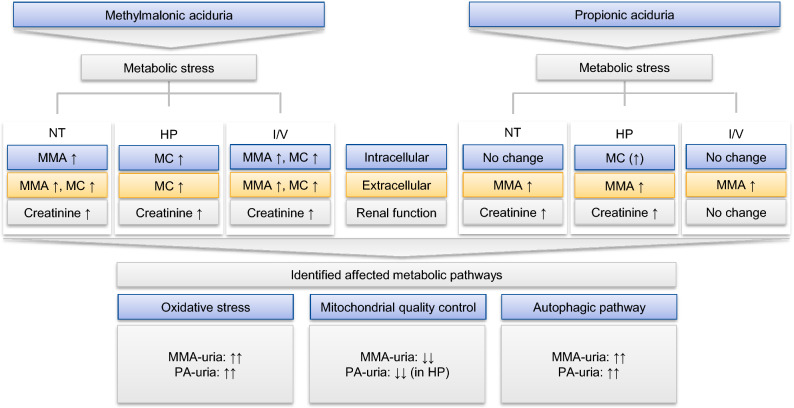
Summary figure. The figure summarizes the main metabolic differences upon the different exposures in MMA-uria and PA-uria. While the disease specific metabolites MMA and MC are present in all conditions in MMA-uria, they are most abundant in HP conditions in PA-uria, where also creatinine levels are highest. Both diseases share elevated oxidative stress levels and the strong activation of the autophagic pathway. Mitophagy is compromised in MMA-uria in all exposures, while a down-regulation in PA-uria was observed only in HP conditions, where also MMA and MC levels were highest. *Blue* intracellular compartment, *yellow* extracellular compartment, *grey* renal function, *MC* methylcitrate, *MMA* methylmalonic acid.

C3 concentrations were elevated in MMA-uria cells under NT conditions. HP and I/V exposure induced a marked increase in C3. The results are in line with our data for renal epithelial cells derived from PA patients^12^, where metabolic challenge with HP and I/V also augmented C3 levels. Lucienne et al.^17^ described a dose-dependent increase of C3 levels in the MMA-uria mouse model following exposure to protein-enriched chows. Creatinine levels were elevated in MMA-uria cells in all conditions. Whereas I/V induced higher creatinine levels in MMA-uria cells, HP led to a further increase in creatinine in PA-uria^12^. This might be explained by different metabolite profiles: In MMA-uria, a rise in intracellular, potentially toxic MMA concentrations is particularly observed in NT and I/V conditions, where creatinine concentrations are highest. In PA-uria, the export of MMA shows the highest concentrations in HP exposure, but intracellular MMA concentrations are low. The more pronounced profile for MMA-uria could hint at a dosage effect for MMA, as one possible mechanism explaining earlier renal involvement (Fig. 9A).

Overall cellular survival is not impaired in MMA-uria cells. Although stressful treatment also affects control cells, MMA-uria cells are more prone to undergo apoptosis, especially in HP and I/V exposure. This might be due to several reasons: First, increased concentrations of MMA and/or MC could be responsible for higher apoptotic rates^18,19^. Second, this could be due to reduced levels of SIRT1, which have been associated with increased apoptotic activity^20,21^ and which are lowered particularly under metabolic stress in our MMA-uria model.

The deleterious effect of elevated ROS levels and dysfunctional mitochondria for the progression of CKD in MMA-uria has been highlighted by different groups^9,10^. We used the glutathione system as indirect read-out for ROS generation. Both HP and I/V load lowered the ratio of reduced to oxidized glutathione suggesting activation of this pathway in MMA-uria. This was comparable to our PA-uria model^12^, pointing at similar CKD-driving mechanisms.

We next investigated, how compromised antioxidant defense might impact on mitochondrial number and mass. Our previous studies^10^ pointed towards an up-regulation of mitochondrial proteins in MMA-uria suggesting a need to compensate for mitochondrial dysfunction. In our current study, HP and I/V exposure even led to a further increase of VDAC in MMA-uria cells, like previously observed in the PA-uria model^12^. VDAC is accepted as a read-out for mitochondrial mass in different tissues^22,23^ and decreased VDAC levels have been associated with mitochondrial depletion and progression of CKD^24^. FACS analysis with MitoSpy, a mitochondrial dye independent of membrane potential, revealed a tendency towards lowered mitochondrial numbers in MMA-uria cells. The loss was evident especially under I/V conditions. Loss of mitochondrial mass has been associated with CKD progression^25^ and might be one factor for CKD progression in MMA-uria. This is of particular interest, since mitochondrial quality control and dynamics are altered in MMA-uria^10^.

The TCA cycle fuels mitochondrial energy generation. Intracellular succinate depletion due to inhibition of TCA cycle enzymes^26^ has been discussed as a potential pathomechanism of energy depletion in organic acidurias. Succinate directly links the TCA cycle to the respiratory chain. Its concentrations were lowered under HP conditions, pointing to reduced substrate supply via this pathway as compared to NT and I/V exposure. Intracellular itaconate concentrations were also low, while itaconate secretion was increased under all treatment conditions. Itaconate is a potent regulator of inflammation. Since patients with MMA-uria suffer from tubulo-interstitial nephritis^1^, itaconate secretion into the extracellular space might be a protective counteracting mechanism. Renal epithelial PA-uria cells showed the same metabolic profile which might point towards similar renal pathomechanisms^12^.

Strikingly, MMA concentrations were highest under NT conditions and did not further increase after HP load. This finding could be explained by the greater availability of HP (25% FBS) as a source of energy which might allow the cells to selectively catabolize amino acids other than those that promote MMA production. Furthermore, FBS might be naturally low in branched-chain amino acids and thus less favorable for MMA production. HP and especially I/V exposure potentiated intracellular concentrations of MC, suggesting that MC might play a more prominent role in renal pathophysiology than MMA at least in metabolically challenging situations. The export of MC was increased in parallel, pointing to a need to remove this potentially toxic metabolite. Of note, MC exposure led to a rise in ammonia concentrations and increased the level of apoptosis^18,19^ in an organoid model of rat brain cells. Elevated MC concentrations have been related to disease progression^27^ and shown to inhibit enzymes linked to energy generation^28^. Intracellular propionate levels were slightly increased by the two exposures. Our results from the PA-uria cells showed reduced MC levels and intracellular MMA concentrations were normal as compared to control cells NT. However, MMA was secreted by PA-uria cells in all conditions accompanied by a rise in creatinine concentrations pointing towards a need for removal. The results point to a slightly different but more pronounced intracellular metabolite profile for MMA-uria (Fig. 9A), which might be responsible for the more severe renal phenotype and matches the clinical observation, that high MMA concentrations are associated with early onset and rapid progression of CKD^3,4^. The obtained results (Fig. 9A) fit well to the metabolic profiles in MMA- and PA-uria patients^1^, except for the observation that PA-uria cells secreted small amounts of MMA that further increased with metabolic challenge. The difference might be explained by the relatively “pure” cellular system and dilution effects masking slight elevations in the human body. Which of these metabolites drive alterations in intracellular signaling and the mode of action remains to be elucidated. Additional hits (like e.g. inflammation^9,29^), might play a role.

We next investigated, if the use of fuels for energy generation might be changed under the different culture conditions. Acylcarnitine profiling has been used as a read-out for fatty acid oxidation (FAO) and disturbed FAO has been associated with the onset and progression of CKD of diverse origin^30^. Our studies revealed accumulation of long- and medium-chain acylcarnitines, indicating a block in the degradation of these highly energetic substrates. Similar findings and lipid droplets have been reported by EM in renal PA-uria cells^12^. Forny et al. detected elevated levels of odd-chain fatty acids in a MMA-uria mouse model^31^. Free carnitine levels are particularly lowered with I/V exposure, which is in line with patients’ data^1^ and points to substrate-specific problems.

Intracellular isoleucine and valine concentrations were increased under I/V stress as a proof of concept. Both HP and I/V exposure led to enhanced intracellular concentrations of glycine and methionine. MMA-uria cells exported indigestible amino acids, again stressful exposure increased export rates. These findings could point towards a rationale for restricting these amino acids in MMA-uria at least for renal tissue.

Mitochondrial quality control is an essential mechanism to maintain a network of functional mitochondria. Our recent studies^10^ suggest a link between defective mitochondrial priming for degradation and renal epithelial damage in MMA-uria. Defective priming seems to be directly correlated to the metabolic burden imposed by HP and I/V load: Both exposures further lowered PINK1 expression, the gate-keeper of mitophagy^32^. PINK1 expression was up-regulated in control cells pointing to the metabolic challenge imposed by HP and I/V. SQSTM1 levels, a well-accepted marker for autophagic processes^33^, were up-regulated under NT conditions and further increased by HP and I/V load pointing to a need to remove dysfunctional mitochondria via bulk autophagy. PINK1 expression was down-regulated in our PA-uria model in HP exposure^12^. In parallel, the highest MMA secretion, MC accumulation and creatinine levels were noticed. A similar pattern was observed for MMA-uria and could point towards a threshold effect for the accumulation of potentially toxic organic acids leading to interference with mitochondrial quality control at a certain concentration (Fig. 9A). This might be one CKD-driving mechanism primarily present in MMA-uria, explaining the milder renal phenotype in PA-uria, where the mechanism is activated upon metabolic stress. The exact mode of action needs further investigation (Fig. 9A).

Mitochondria are highly dynamic organelles constantly rearranging their networks via mitochondrial fission and fusion. Fission is a sentinel hub, allowing cells to sense and respond to (metabolic) external stimuli and is finally executed by Drp1^34^. Our results revealed an up-regulation of Drp1 expression and thus a strong tendency towards fission in MMA-uria cells. Fusion, allowing exchange between mitochondrial networks in times of cellular homeostasis, was down-regulated as evidenced by lowered levels of OPA1 in MMA-uria cells.

Increased mitochondrial fission in PINK1-deficient cells is a striking finding, since PINK1 is one possible activator of Drp1-mediated fission. However, similar findings have been obtained in other CKD models^25^. Furthermore, Drp1 is the target of different pre- and posttranslational modifications (e.g. calcium calmodulin dependent kinase, cyclin B-cyclin-dependent kinase)^35^ and could be activated by alternative pathways.

Although disease mechanisms are different, alterations in mitochondrial dynamics have been discussed in the context of diabetes^36^ and could point to a more general pathomechanism for CKD. Promotion of mitochondrial fusion by anti-inflammatory drugs (leflunomide) has been investigated as treatment option in mitochondrial dysfunction^37^. This is of particular interest in the context of MMA-uria, since inflammation has been discussed as another potential disease driving mechanism of CKD.

Sirtuins are NAD^+^-coupled deacetylases exerting cytoprotective effects by governing anti- apoptotic, -fibrotic and -inflammatory signaling pathways. We investigated SIRT1 expression, which is found in proximal and distal tubular cells^13^. HP and I/V exposure led to a significant loss of SIRT1 expression in MMA-uria cells, while control cells were able to maintain SIRT1 levels. SIRT1 plays an important role in the governance of lipid and glucose turn over and nutrient deprivation induces SIRT1 activation^38,39^. Besides other modifiers, SIRT1 can be down-regulated by activation of autophagy, a pathway which is highly active in our system^38^. Increased mitochondrial fission has been associated with increased acylation of sterol regulatory element-binding protein 1 (SREBP1) suppressing SIRT1 signaling^40^. In turn, elevated sirtuin levels have been linked to silencing of Drp1 via phosphorylation^41^. A similar mechanism could be the reason for our observations. Furthermore, SIRT1 is an important activator of quality control pathways in the kidney. The observed loss of PINK1 levels and the increased rate of apoptosis in MMA-uria cells could thus be due to low SIRT1 levels^13,39,41,42^. Sirtuins are targetable by pharmacological compounds such as resveratrol^13^ and could be evaluated as potential therapeutic targets in a next step.

PGC-1α -levels were reduced in MMA-uria cells. PGC-1α belongs to a family of transcription factors and is involved in mitochondrial biogenesis and homeostasis^43,44^. Besides others, it is controlled by environmental factors and the sirtuin family. SIRT1 activity has been correlated to high PGC-1α levels which are renal protective^44^. Reduction in PGC-1α levels have been associated with fragmented mitochondria, elevated Drp1 levels and reduced mitochondrial fusion proteins^44–46^. The lowered PGC-1α levels might explain the tendency to a lowered mitochondrial mass in MMA-uria and a need to up-regulate mitochondrial proteins. However, the function of PGC-1α is highly cell specific and the number of possible interacting factors numerous. Further studies will be needed to investigate possible interactions.

Quite a number of inborn errors of metabolism present with CKD as long-term complication: Altered OXPHOS activities and increased mitochondrial mass have been reported in a rat model for glutaric aciduria type 1^47^. In Fabry disease, impaired mitochondrial homeostasis and altered autophagic flux have been described^48,49^. Metabolomic studies in the plasma of glycogen storage disease type 1 patients revealed abnormalities in fuel utilization and energy metabolism^50^. These observations point towards mitochondrial impairment as a joint feature and highlight the mitochondrion as fate deciding organelle in different disease entities.

A potential limitation of our study is the use of FCS at different concentrations (10% for NT, 25% for HP and 5% for I/V exposition) during metabolic challenges. Besides being a mixed source of diverse proteins, FCS is also rich in lipids and growth factors, which might exert additional effects (including dilution bias) we cannot correct for.

In summary, we investigated the impact of metabolic stressors on mitochondrial energy metabolism and homeostasis in a renal epithelial model for MMA-uria. Our study supports the idea, that altered mitochondrial quality control and dynamics favor altered mitochondrial homeostasis and are related to CKD in MMA-uria and other organic acidurias. Besides MMA, metabolic challenge suggested MC as another potentially relevant toxic metabolite for CKD progression. If and how the toxic metabolites interfere with the identified signaling pathways and thus hold responsive for the observed mitochondrial imbalances remains to be elucidated. Targeting these pathways by small molecules^36,37^ might serve as potential new treatment options to ameliorate CKD in MMA-uria.

## Materials and methods

### Cell culture

Human renal epithelial cells from healthy controls (n = 3) and MMA-uria patients (n = 3) deficient in methylmalonyl-CoA mutase (*MUT*^0^) were cultured as previously described^10,28^. All patient cell lines have a residual MUT activity of 1–2%^10^. Cells were collected from spot urine, taken in primary culture and immortalized after one passage using electroporation and a SV-40 vector^10,28^. The cells were used between passage 4 and 10, all starting at passage 4. Exposure time was 7 days for each condition. During the experiment, the cell culture medium was changed every 48 h. The assays on cell culture medium were performed on the very last supernatant before harvest after 48 h of incubation. Cellular growth was comparable for the cell lines in the different treatment conditions. The cells were harvested at 90% confluency. The DMEM-based cell culture medium was composed of (1) high glucose medium (4.5 g/100 mL, Thermo Fisher Scientific), 10% fetal calf serum (FCS, Biochrome), 5% Pen/Strep (100 U/mL penicillin, Thermo Fisher Scientific) for NT conditions, or (2) low glucose medium (1 g/100 mL, Thermo Fisher Scientific) supplemented with (a) 25% FCS (HP) and PenStrep or (b) 1000 µM isoleucine/3000 µM valine, 5% FCS and PenStrep (100 U/mL penicillin) for 7 days. The concentrations of vitamin B_12_, isoleucine (0.8 mM), leucine (0.8 mM), threonine (0.8 mM) and valine (0.8 mM) were identical in all used DMEM standard media as was the concentration of all other amino acids and buffer substances.

### Statistical analysis

The main goal of this study was to investigate the effect of each intervention in comparison to the Control NT group. We applied two-way ANOVA for comparison of different categorical independent variables on a dependent variable and one-way ANOVA with treatment as main factor (six levels: Control NT, MMA NT, Control HP, MMA HP, Control I/V and MMA I/V) for comparisons between two groups. If factor treatment was significant, we performed multiple comparison tests between each treated group and the Control NT group (fives comparisons: Control NT vs*.* MMA NT, Control NT vs*.* Control HP, Control NT vs*.* MMA HP, Control NT vs*.* Control I/V and Control NT vs*.* MMA I/V). We adjusted the p-values with Bonferroni correction. The asterisk provided refer to control NT conditions for the sake of tidyness. The results are expressed as mean ± standard error of mean (SEM). Each figure legend provides the sample size. The datasets are representative of at least three independent repeats. Statistical analyses were performed using GraphPad Prism software. Statistical significance is indicated as *P* ≤ 0.05 (*), *P* ≤ 0.01 (**), *P* ≤ 0.001 (***), *ns*: not significant.

### Analysis of amino acids

Amino acid concentrations were determined using a Biochrom 30 amino acid analyzer (Biochrome) according to a previously published protocol^28,49^. Briefly, after trypsinization of cells grown in T75 cell culture flasks, cells were rinsed with PBS and immediately frozen at − 80 °C. Cells were resuspended with 250 µL of lysis buffer (PBS supplemented with 1% protease inhibitor cocktail) and freeze-thawed in dry ice followed by a sonication step (three cycles). After centrifugation (9447×*g* for 15 min at 4 °C) the lysates were analyzed according to the routine procedures for amino acid analysis used at the Laboratory of Clinical Biochemistry and Metabolism at Freiburg university. Protein concentration was determined by a BCA assay (Pierce, Thermo Fisher Scientific).

### Antibodies used for immuno-blotting

anti-PINK1, #23707, rabbit polyclonal, (Abcam); anti- SQSTM1, MBL PM045, rabbit polyclonal, (MBL International Corporation); anti-VDAC, #4661, rabbit monoclonal (Cell signaling Technology); anti-DRP1, rabbit monoclonal, #8570 (Cell signaling Technology); anti-OPA1 rabbit monoclonal, #80471 (Cell signaling Technology); anti-SIRT1, rabbit polyclonal, #2310, (Cell signaling Technology).

### Immuno-blotting

Cells were lysed in RIPA buffer and sonicated. The protein concentration was determined using a BCA assay (Pierce, Thermo Fisher Scientific). Samples were loaded at 20 µg/lane. Separation of protein was performed by SDS-PAGE. Blotting was performed using nitrocellulose membranes. 5% non-fat milk (1706404, Bio-Rad Laboratories) was used for blocking after incubation with the primary (4 °C, 24 h) and the peroxidase labelled-secondary antibody (18 °C, 2 h), membranes were evaluated using chemiluminescence (WBKLS0050, Millipore, Life technologies). Image J software (imagej.nih.gov^51^) was used to quantify the density of each signal using γ-tubulin for protein normalization.

### Quantitative profiling of intra- and extracellular metabolites by liquid chromatography and mass spectrometry (LC–MS/MS)

Whole cell lysates were aliquoted for polar metabolite and acylcarnitine profiling and total protein concentration, and stored at − 80 °C until the day of sample preparation. Cell culture media were cleared by centrifugation at 10,000×*g*, 4 °C, for 10 min to remove dead cells and debris, and stored at − 80 °C until further analysis. Sulfur-containing metabolites as well as creatinine, S-adenosylmethionine and S-adenosylhomocysteine were determined according to a previously published procedure^52–54^. Lactate, TCA and glycolysis intermediates and other organic acids, and folates were determined as described in previous work^53^. We used ^13^C_5_-itaconate (Sigma), ^13^C_1_-lactate (Sigma), D_3_-methylmalonic acid (CDN isotopes), and D_4_-succinate (Sigma) as internal standards. D_3_-methylmalonate was used as a generic internal standard when isotopes were not available. Amino acids were determined using a previously described protocol^55^, with modifications. Briefly, 20 µL of sample were injected onto a X-terra^®^ C18 chromatography column (5 µm, 3.9 × 150 mm, Waters) and the metabolites separated at a flow rate of 0.5 mL/min of solvent A (0.1% formic acid in water) and solvent B (0.1% formic acid in MeOH) according to the following gradient: 0–0.50 min (2% B), 0.5–5.5 min (20% B), 5.5–7.5 (80% B); 7.5–8.0 min (80% B), 8.0–8.5 min (2% B), 8.50–15 min (2% B). A commercially available standardized amino acid mixture was utilized to generate a calibration curve for amino acids (Amino acid standards, physiological, Sigma, Nr. A9906-10ML). Calibration curves for all other metabolites were prepared from individual stock solutions prepared in house. Quantitation accuracy was examined by monitoring homocysteine and methylmalonic acid concentrations in an external quality control, namely, the Control Special Assays in Serum, European Research Network for the evaluation and improvement of screening, diagnosis, and treatment of Inherited disorders of Metabolism (ERNDIM) IQCS, SAS-02.1 and SAS-02.2 from MCA Laboratories, Winterswijk, Netherlands. For all other metabolites, quantitation trueness was tested by examining metabolite concentrations in plasma from a previously validated sample isolated from a healthy control individual with respect to standard reference ranges, using the same calibration curves and LC–MS/MS running conditions. Data for polar metabolite profiling were acquired on a Sciex 6500 + ESI-tripleQ MS/MS (AB Sciex Germany GmbH, Darmstadt, Germany) coupled with an ultra-performance liquid chromatography (Nexera, Shimadzu).

Acylcarnitines were isolated by methanol extraction starting with 50 µL of tissue homogenate. Samples were processed according to standard operating practices for the determination of acylcarnitine profiles in plasma (Laboratory of Clinical Biochemistry and Metabolism, Center of Metabolism Freiburg). Acylcarnitines were determined via flow-injection analysis into a Sciex 5500 + ESI-tripleQ MS/MS (AB Sciex Germany GmbH, Darmstadt, Germany). Graphical analysis and comparison of acylcarnitine abundance was simplified by pooling the species according to their acyl group chain length, as follows: free carnitine (C0), short chain (C2–C5), medium chain (C6–C10) and long chain (C12–C18).

All metabolite concentrations were normalized by the total concentration of protein in the cell lysate, determined using the BCA assay (Pierce, ThermoFisher Scientific). Metabolites concentrations (µM) were divided by the concentration of protein (mg/mL) to obtain nmol/mg protein.

#### Signal processing and data analysis

Quantification of metabolites was carried out with Analyst^®^ 1.7.2 software, 2022 AB Sciex.

### Collection of conditioned culture medium for metabolomic analysis

An aliquot of 0.25 mL of conditioned culture medium was transferred into a clean 1.5 mL Eppendorf tube and centrifuged at 10,000×*g*, 4 °C, for 10 min to remove dead cells and debris. The cleared supernatant was transferred into a clean, safe-lock 1.5 mL Eppendorf tube and stored at − 80 °C until metabolomic analysis. All metabolite determinations employed 20 µL of cleared conditioned culture medium, which was subjected to the necessary sample preparation protocol^52,53^.

### Fluorescence activated cell sorting

To determine mitochondrial mass as well as lytic cell death and apoptosis, the cells were analysed by Fluorescence Activated Cell Sorting (FACS). After detaching, both live and dead cells were counted by trypan blue staining in a Neubauer chamber. For subsequent stainings, the cells were resuspended in FACS buffer (1 × PBS + 2% FCS) and cell count was adjusted to 250k cells per well in a v-bottom 96-well plate. For determining cell death, cells were stained for Annexin V and 7AAD according to the manufacturer’s protocol (Biolegend, cat# 640926). Separate samples were stained with MitoSpy Green FM (Biolegend, cat# 424805) to determine the mitochondrial mass. Cells were incubated with MitoSpy dilution (1:10,000 in FACS buffer) for 30 min at 37 °C and subsequently incubated with DAPI solution (1:10,000 in FACS buffer), followed by washing with FACS buffer. Samples were analysed on a FACSFortessa cytometer and FACSDiva software (BD Biosciences).

### Statistical analysis for FACS

For analysis of the Annexin V/7AAD staining, the percentage of cells in each quadrant was used as a read-out. To determine the mitochondrial mass from the MitoSpy signal, the geometric mean fluorescent intensity was used for further statistical analysis.

### A patient consent statement/details of ethics approval

All individuals gave their informed consent. The study was carried out following the criteria of the Helsinki Declaration of 1975, as revised in 2000. The generation of the cell lines was approved by the ethical committee of Heidelberg university (ethical vote: S-074/2008). All research was performed in accordance with the research guidelines of Freiburg university.

### Documentation of approval from the Institutional Committee for Care and Use of Laboratory Animals (or comparable committee)

No animal models have been used in this study.

## Supplementary Information

Supplementary Information.

Supplementary Figures.

## Data Availability

The datasets reported are available upon reasonable request to the corresponding author.
