# Supplementary material for: The impact of metabolic stressors on mitochondrial homeostasis in a renal epithelial cell model of methylmalonic aciduria
Source: Sci Rep. 2023 May 11;13:7677. doi: 10.1038/s41598-023-34373-8 (PMC10175303; doi:10.1038/s41598-023-34373-8)
Supplement: Supplementary file 1 — Supplementary Information. [file 41598_2023_34373_MOESM1_ESM.docx]

*Supplementary Data*

*Supplementary table 1*

***(A)***

| *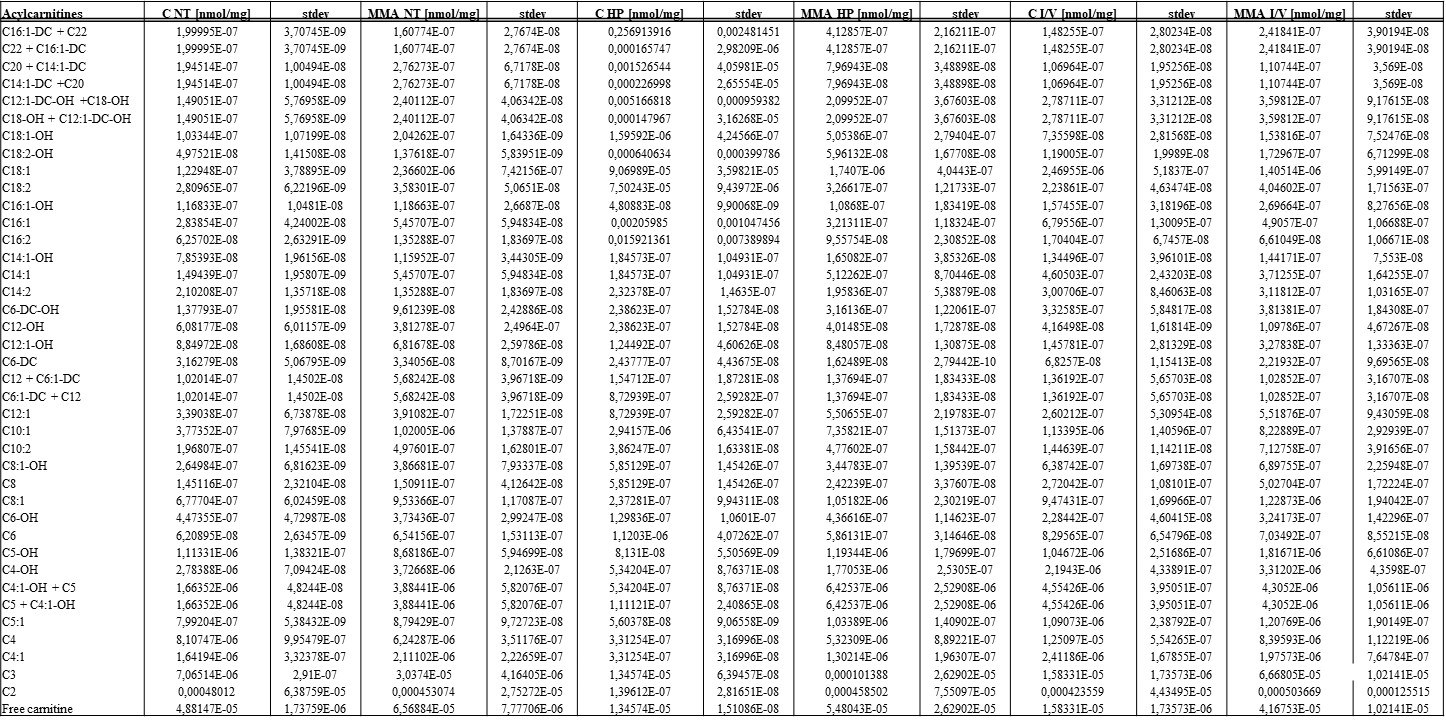* |
| --- |
| *Absolute concentrations of acylcarnitines*  Supplementary table 1 showing the absolute concentrations of intracellular acylcarnitines; n=9 per group; NT: normal treatment; HP: high protein; I/V: isoleucine/valine exposure. Average in [nmol/mg protein]. Stdev: standard deviation. |

*Supplementary table 2*

| **(A)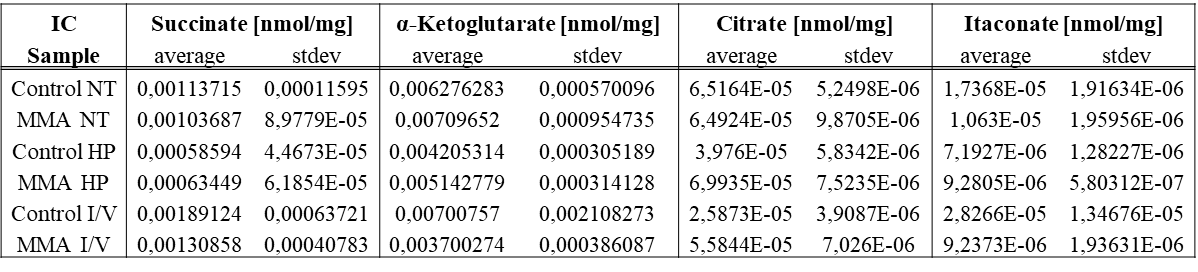** |
| --- |
| 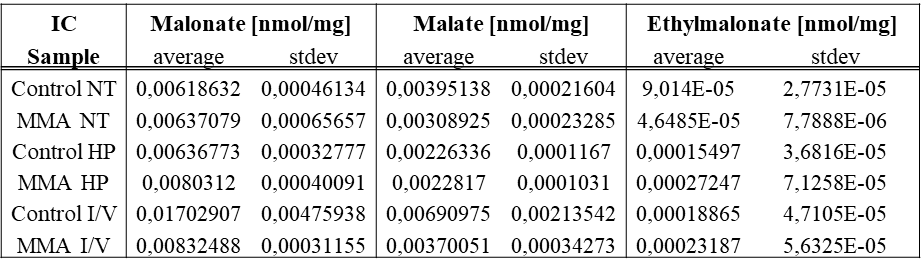 |
| **(B)**  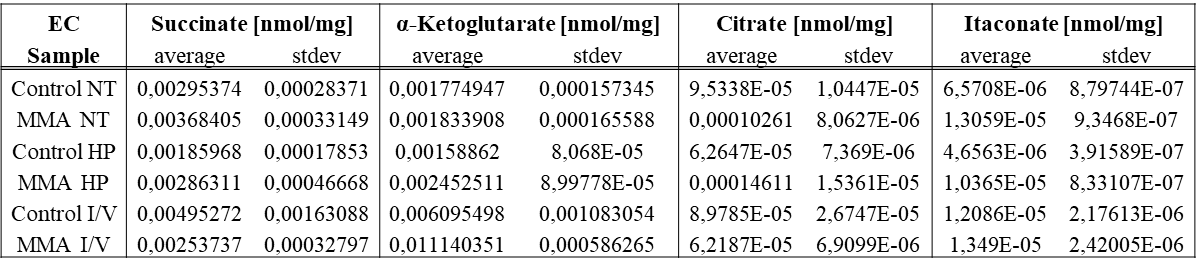 |
| 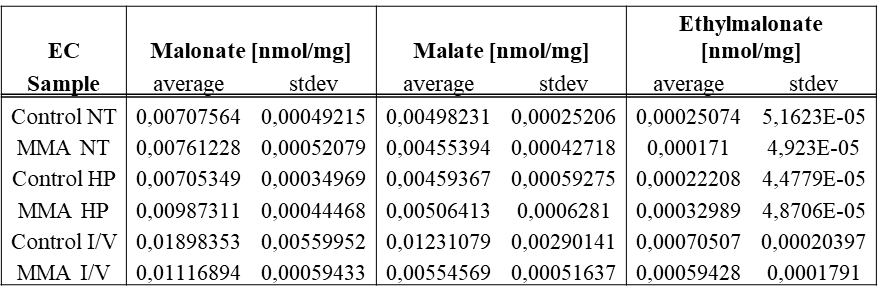 |
| *Absolute concentrations of intra- and extracellular intermediate metabolites*  Supplementary table 2 showing **(A)** intracellular (IC) and **(B)** extracellular (EC) intermediate metabolites; n=9 per group; NT: normal treatment; HP: high protein; I/V: isoleucine/valine exposure. Average in [nmol/mg protein]. Stdev: standard deviation. |

*Supplementary table 3*

| **(A)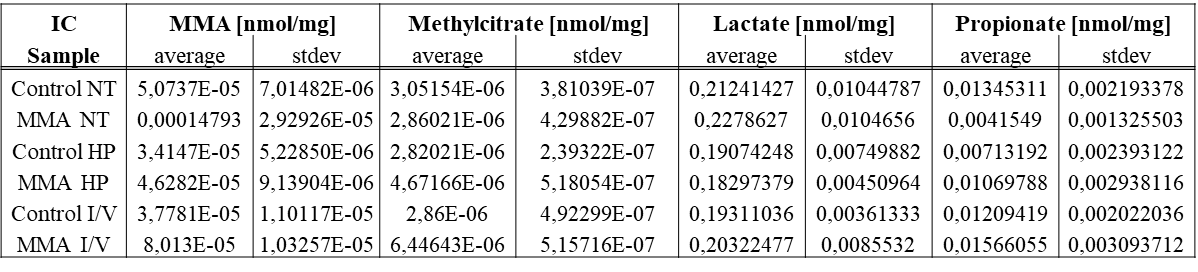** |
| --- |
| **(B)**  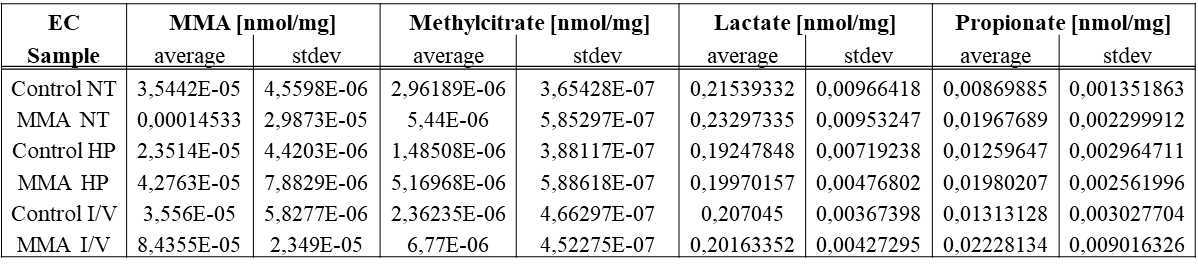 |
| *Absolute concentrations of intra- and extracellular MMA associated metabolites*  Supplementary table 3 showing **(A)** intracellular (IC) and **(B)** extracellular (EC) MMA-uria associated metabolites; n=9 per group; NT: normal treatment; HP: high protein; I/V: isoleucine/valine exposure. Average in [nmol/mg protein]. Stdev: standard deviation. |

*Supplementary table 4*

| **(A)**  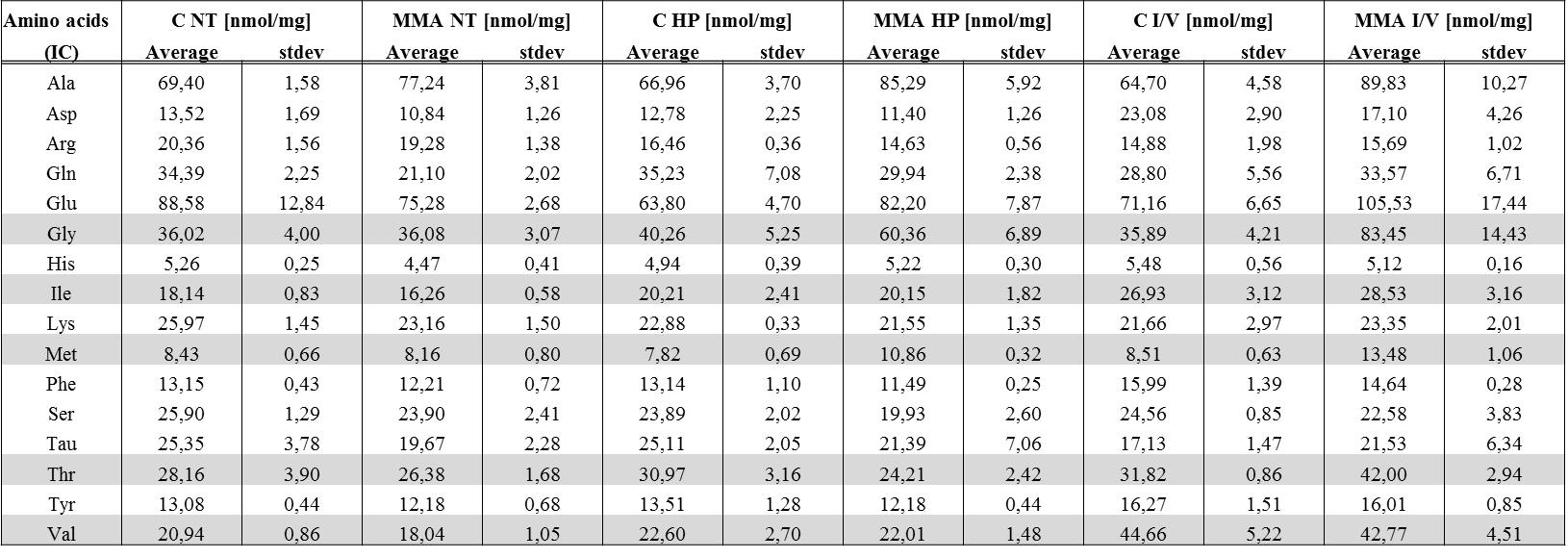 |
| --- |
| **(B)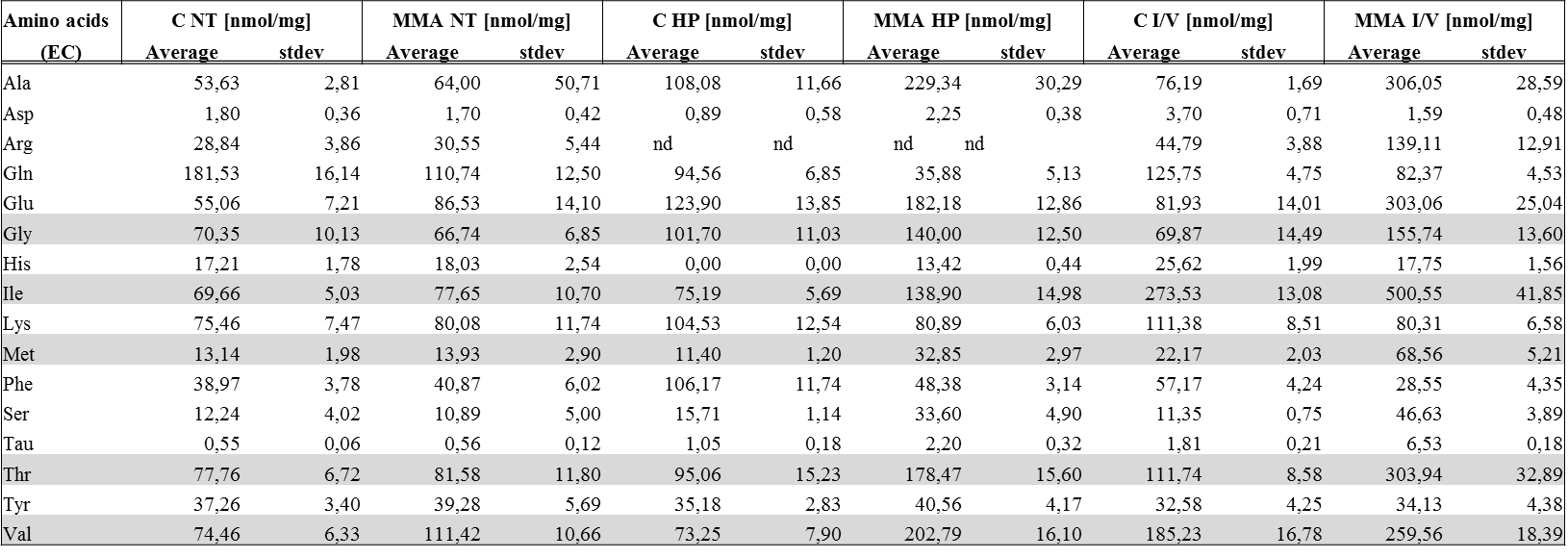** |
| Supplementary table 4 depicting the absolute concentrations of intracellular **(A)** and extracellular amino **(B)** acids; n=9 per group. The characteristic amino acids are highlighted. Ala: alanine; Asp: asparagine; Arg: arginine; Gln: glutamine; Glu: glutamate; Gly: glycine; His: histidine; Ile: isoleucine; Lys: lysine; Met: methionine; Phe: phenylalanine; Ser: serine; Tau: taurine; Thr: threonine; Tyr: tyrosine; Val: valine. NT: normal treatment; HP: high protein; I/V: isoleucine/valine exposure. Average in [nmol/mg protein]. Stdev: standard deviation.  *Supplementary figure legends*  Supplementary figure 1: **(A-G)** shows representative uncropped and unprocessed versions of immune-blots presented in the study.  Supplementary figure 2: **(A)** *Gating strategy* Cells are identified by excluding debris in the FSC vs. SSC gate, followed by excluding doublets in the FSC-A vs. FSC-H gate**. (B)** *Lytic and apoptotic cell death in control and MMA samples.* Double-negative, viable cells (Q4), Annexin V-positive/7AAD-negative, early apoptotic cells (Q1) and double-positive, late apoptotic cells (Q2). |
|  |
