# Supplementary material for: The impact of metabolic stressors on mitochondrial homeostasis in a renal epithelial cell model of methylmalonic aciduria
Source: Sci Rep. 2023 May 11;13:7677. doi: 10.1038/s41598-023-34373-8 (PMC10175303; doi:10.1038/s41598-023-34373-8)

Supplementary figure 1

(A) VDAC

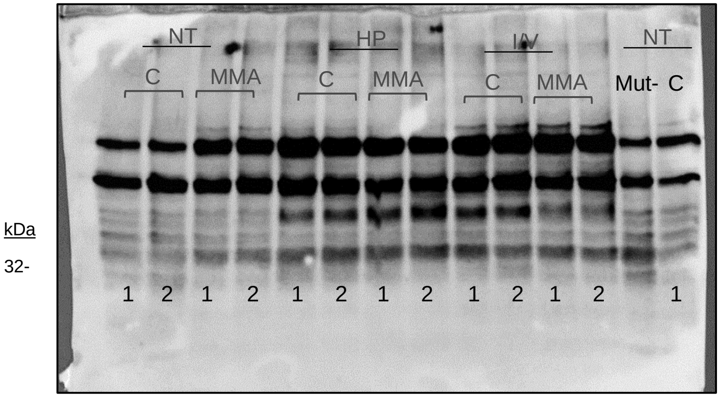

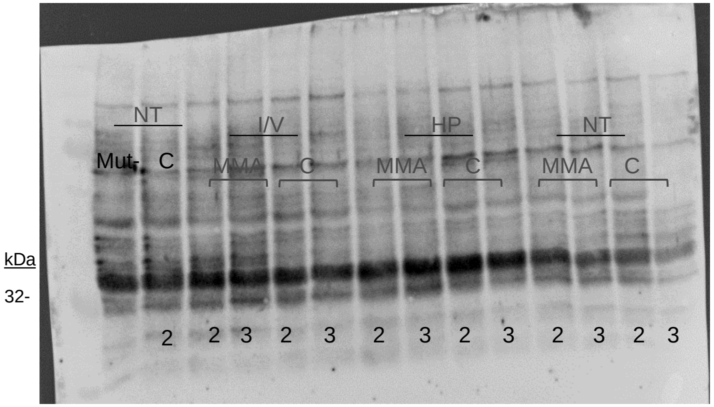

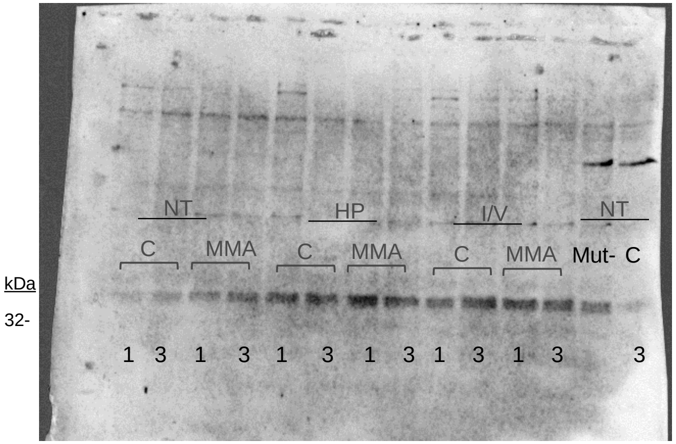

Supplementary figure 1

(B) SQSTM1

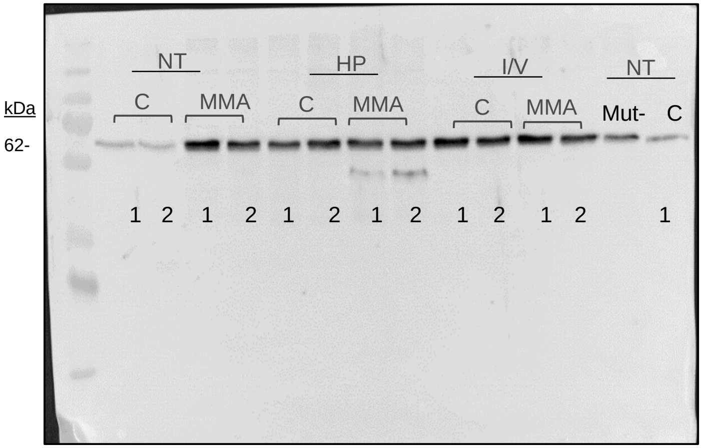

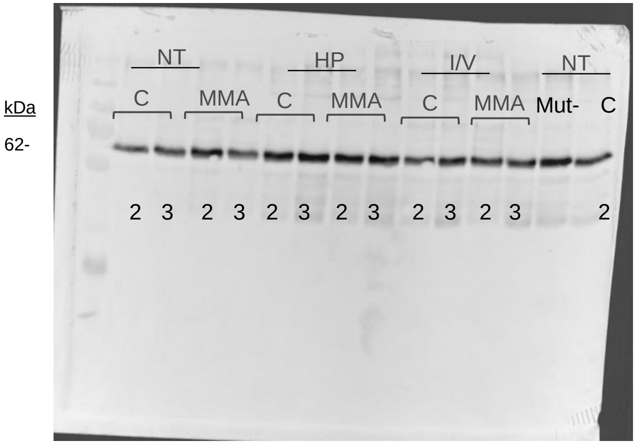

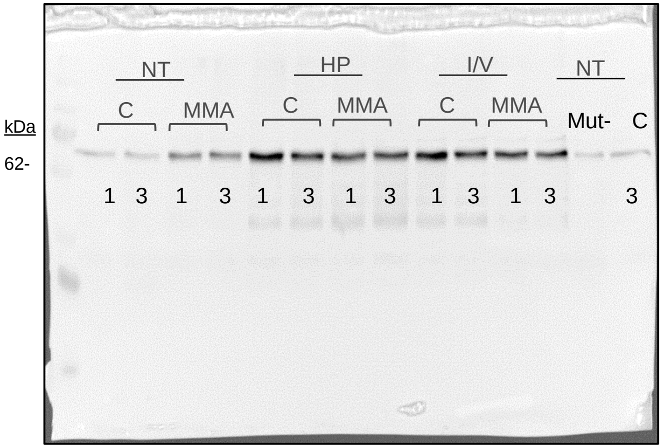

Supplementary figure 1

(C) PINK1

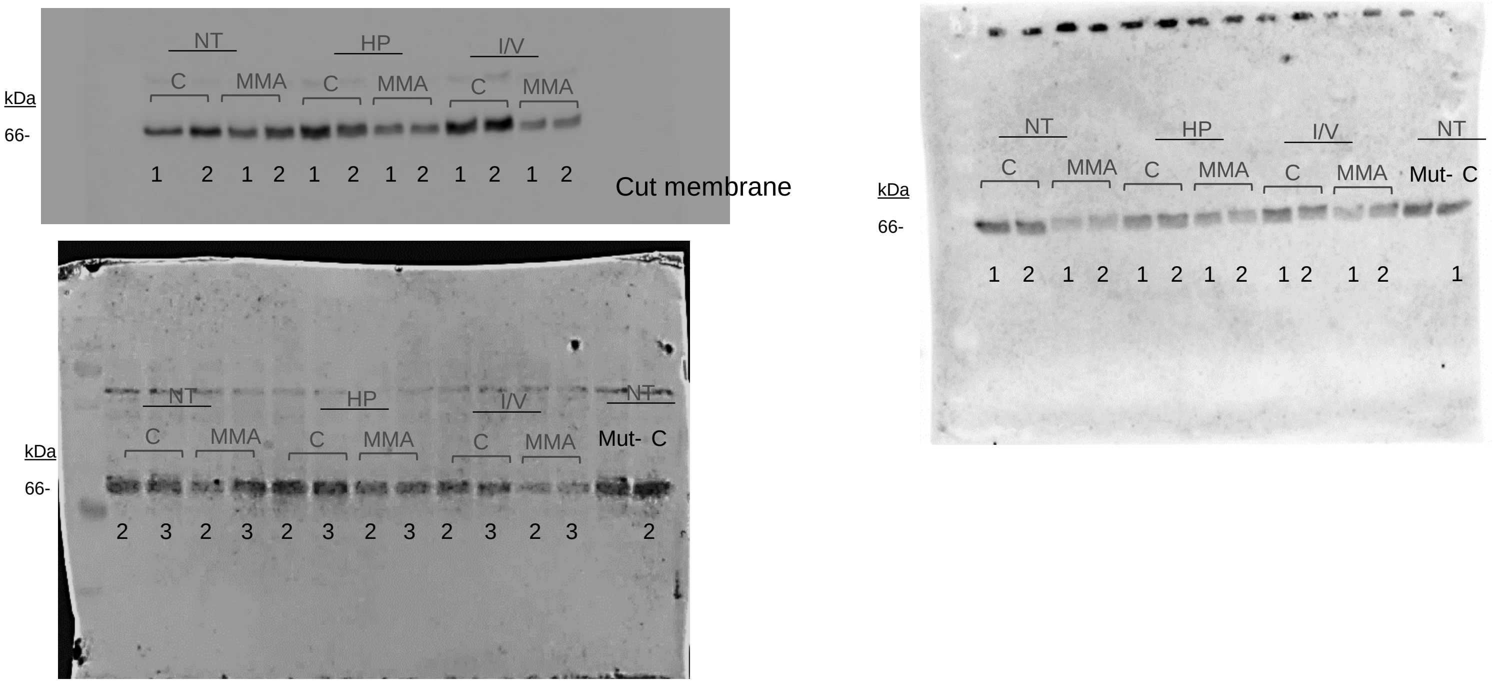

Supplementary figure 1

(D) Drp1

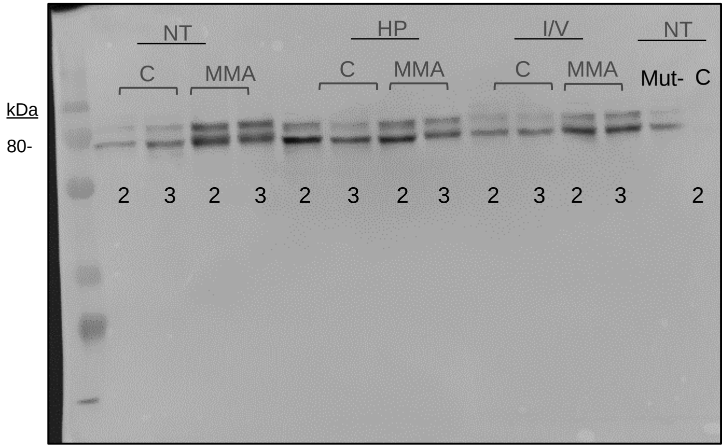

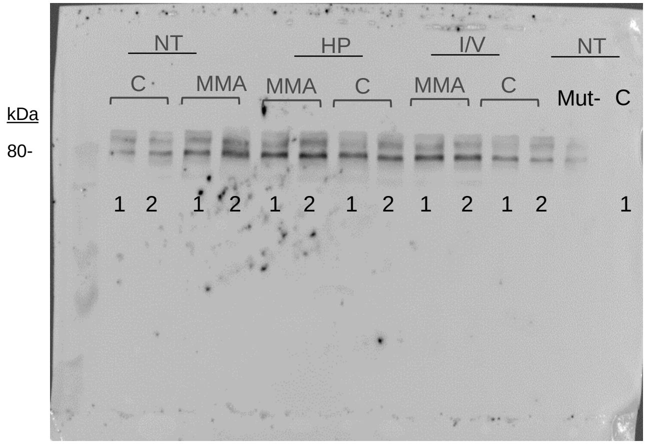

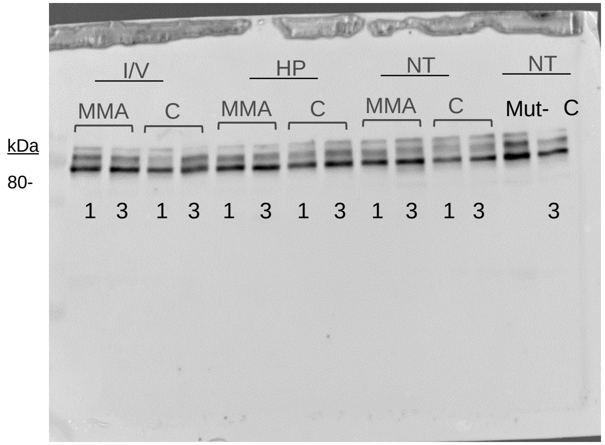

Supplementary figure 1

(E) SIRT1

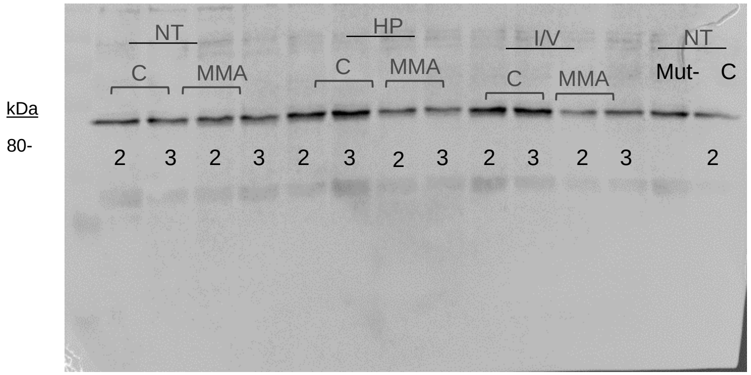

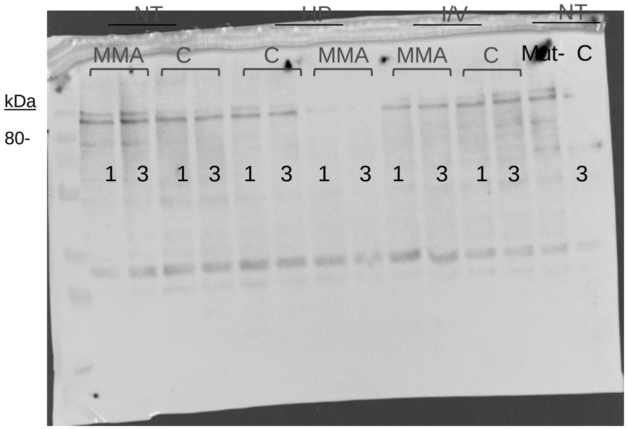

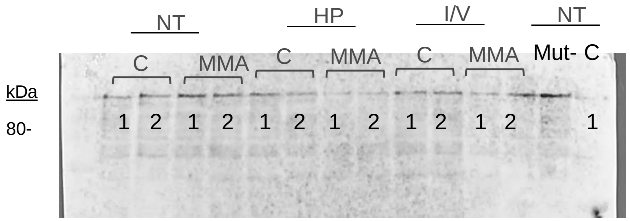

Cut membrane

Supplementary figure 1

(F) OPA1

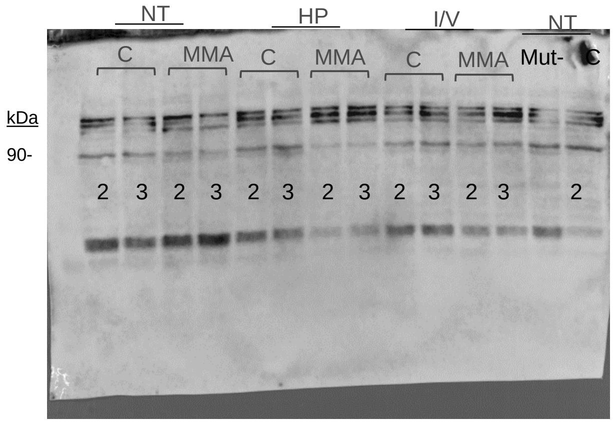

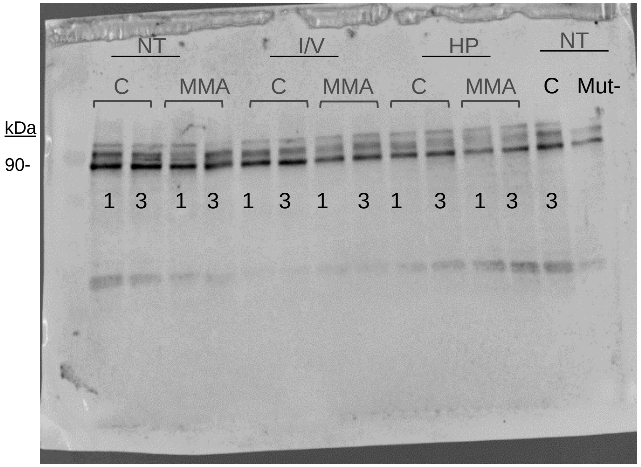

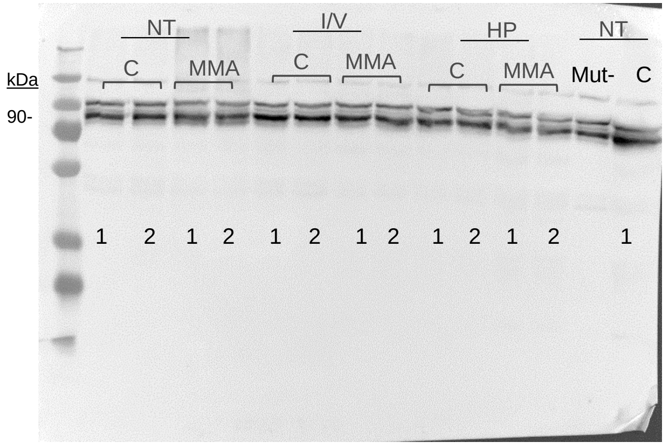

Supplementary figure 1

(G) PGC1 alpha

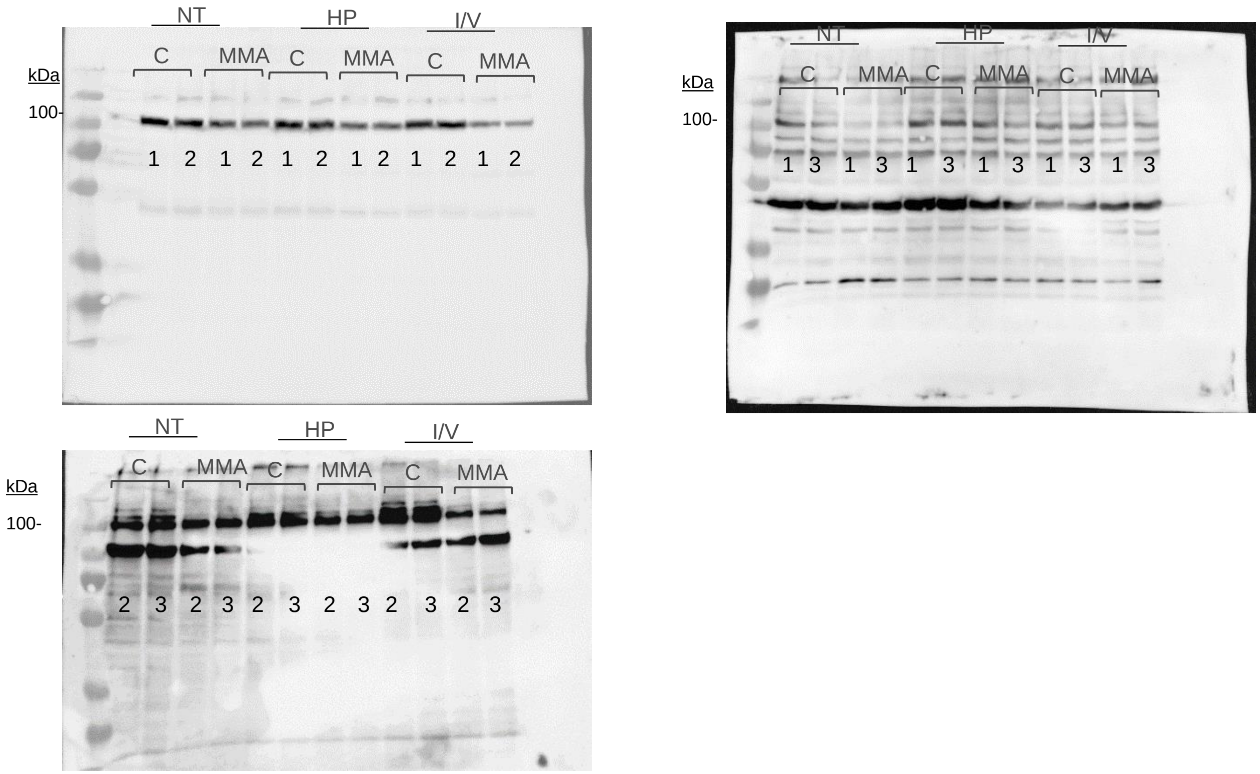

Supplementary figure 1

(H) Tubulin

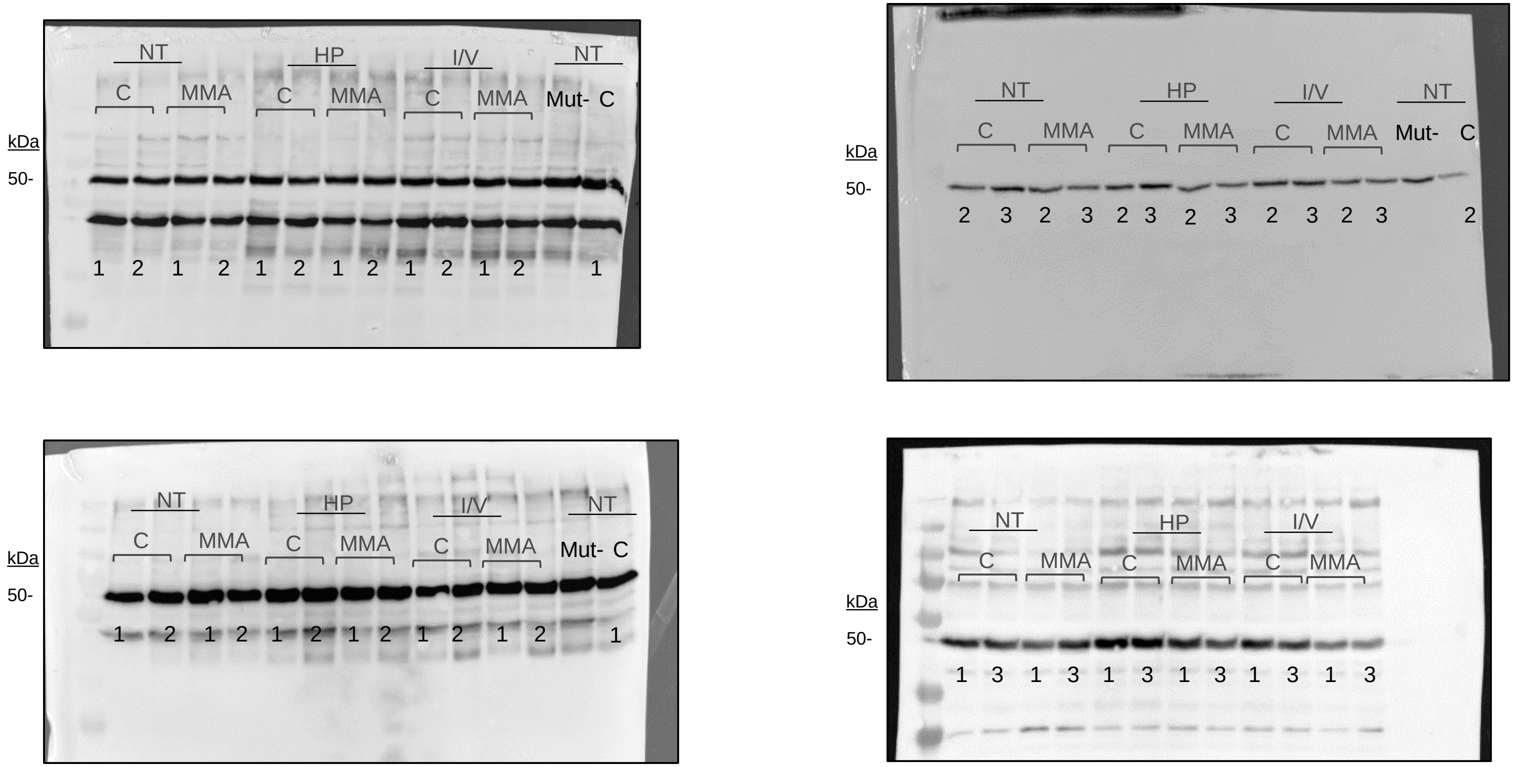

Supplementary figure 2

(A) Gating strategy

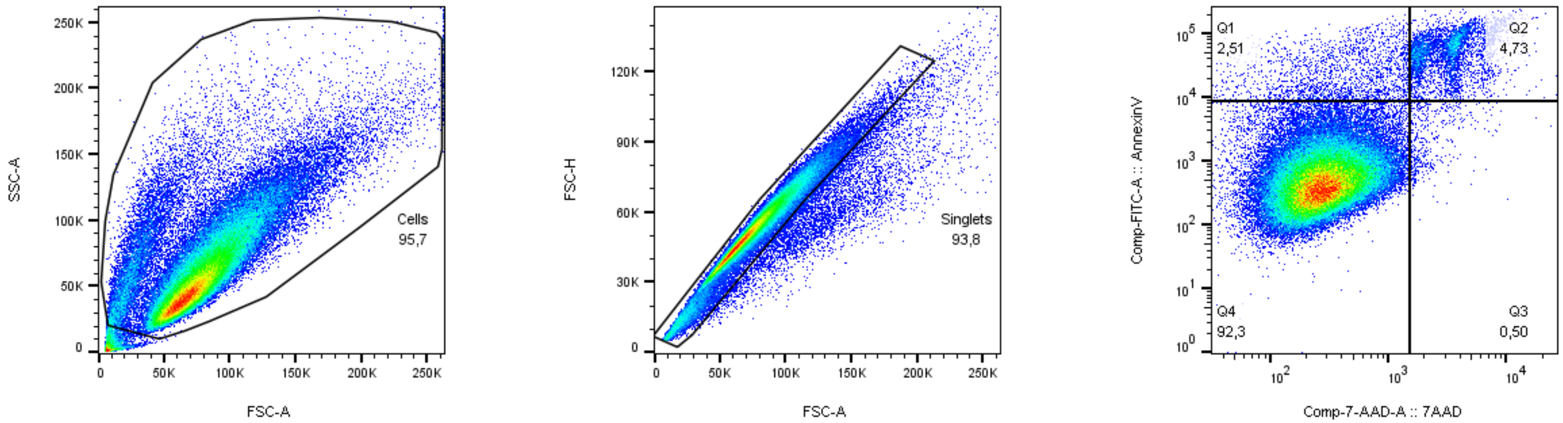

Supplementary figure 2

(B) AnV-7AAD summed data

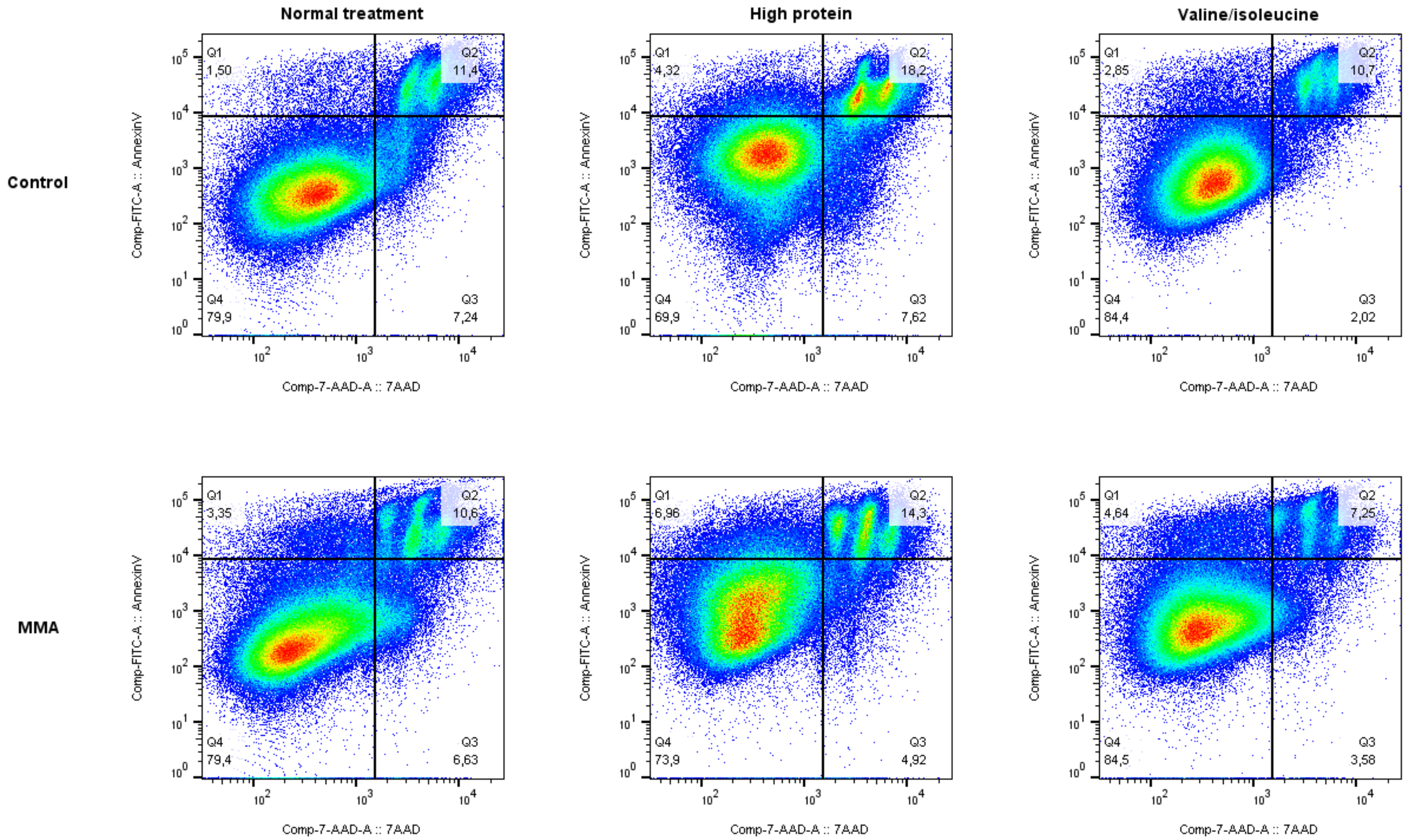

Supplement: Supplementary file 2 — Supplementary Figures. [file 41598_2023_34373_MOESM2_ESM.pdf]
